# Hypothermia amongst neonatal admissions in Kenya: a retrospective cohort study assessing prevalence, trends, associated factors, and its relationship with all-cause neonatal mortality

**DOI:** 10.3389/fped.2024.1272104

**Published:** 2024-03-27

**Authors:** John Wainaina, Morris Ogero, Livingstone Mumelo, Kefa Wairoto, George Mbevi, Timothy Tuti, Paul Mwaniki, Grace Irimu, Mike English, Jalemba Aluvaala, The Clinical Information Network Author Group, Dolphine Mochache

**Affiliations:** ^1^ Health Services Unit, KEMRI-Wellcome Trust Research Programme, Nairobi, Kenya; ^2^ Infectious Disease Epidemiology, London School of Hygiene and Tropical Medicine, University of London, London, United Kingdom; ^3^ Department of Paediatrics and Child Health, University of Nairobi, Nairobi, Kenya; ^4^ Nuffield Department of Clinical Medicine, Oxford, United Kingdom

**Keywords:** newborn, inpatient, hypothermia, mortality, warm chain

## Abstract

**Background:**

Reports on hypothermia from high-burden countries like Kenya amongst sick newborns often include few centers or relatively small sample sizes.

**Objectives:**

This study endeavored to describe: (i) the burden of hypothermia on admission across 21 newborn units in Kenya, (ii) any trend in prevalence of hypothermia over time, (iii) factors associated with hypothermia at admission, and (iv) hypothermia's association with inpatient neonatal mortality.

**Methods:**

A retrospective cohort study was conducted from January 2020 to March 2023, focusing on small and sick newborns admitted in 21 NBUs. The primary and secondary outcome measures were the prevalence of hypothermia at admission and mortality during the index admission, respectively. An ordinal logistic regression model was used to estimate the relationship between selected factors and the outcomes cold stress (36.0°C–36.4°C) and hypothermia (<36.0°C). Factors associated with neonatal mortality, including hypothermia defined as body temperature below 36.0°C, were also explored using logistic regression.

**Results:**

A total of 58,804 newborns from newborn units in 21 study hospitals were included in the analysis. Out of these, 47,999 (82%) had their admission temperature recorded and 8,391 (17.5%) had hypothermia. Hypothermia prevalence decreased over the study period while admission temperature documentation increased. Significant associations were found between low birthweight and very low (0–3) APGAR scores with hypothermia at admission. Odds of hypothermia reduced as ambient temperature and month of participation in the Clinical Information Network (a collaborative learning health platform for healthcare improvement) increased. Hypothermia at admission was associated with 35% (OR 1.35, 95% CI 1.22, 1.50) increase in odds of neonatal inpatient death.

**Conclusions:**

A substantial proportion of newborns are admitted with hypothermia, indicating a breakdown in warm chain protocols after birth and intra-hospital transport that increases odds of mortality. Urgent implementation of rigorous warm chain protocols, particularly for low-birth-weight babies, is crucial to protect these vulnerable newborns from the detrimental effects of hypothermia.

## Background

Mortality risk in children is highest during their first month after birth, where 17 for every 1,000 live-born babies die globally (1, 2). A third of these deaths happen on the first day and two-thirds by the first week of life (1). Most of these deaths (98%) happen in Low-and-Middle-Income Countries (LMICs) (2, 3). In Kenya, a study from 14 public hospitals reported that neonates accounted for 46% of all pediatric admissions (excluding surgical admissions) and 66% of all pediatric mortality in those aged 0–13 years (4).

Early essential newborn care is required to support the transition from an intrauterine to an extrauterine environment (5). It includes assessing breathing for resuscitation need within first minutes to one hour of birth, keeping the baby warm (thermal care), delayed cord clamping, and early initiation of breastfeeding (6–8).

Thermal care aims to maintain normal body temperature (36.5°C–37.5°C). This requires a package of interventions known as the warm chain which includes immediate drying and skin-to-skin contact, and delayed bathing (7, 9, 10). Essential interventions including maintaining warmth has the potential to avert up to 75% of neonatal deaths (10). Neonatal hypothermia has been associated with a four-fold increase in the risk of mortality (RR, 4.66; 95% CI, 3.47–6.24) (11). In addition, hypothermia is associated with the development of metabolic acidosis, jaundice, respiratory distress syndrome (RDS), and poor feeding (12, 13).

Globally, data on neonatal hypothermia is predominantly from hospitals and prevalence has ranged between 32% to 85% soon after birth (14–16). A systematic review that included 12 studies from 24 hospitals in three countries in East Africa reported the average neonatal hypothermia prevalence to be 57.2% (95% CI 39.5, 75.0) (15). In Kenya, only two articles by Ocharo et al. (2021, *unpublished*) and Nyandiko et al. (2021) from relatively small populations and single hospitals are available reporting hypothermia at admission in 67%(180/268) and 74% (274/372) of all Newborn Unit (NBU) admissions respectively (17, 18).

Factors associated with neonatal hypothermia include prematurity, low birthweight, intrauterine growth restriction, low APGAR scores/birth asphyxia, and hypoglycemia (19–21). In addition, geographically associated factors include birthing area environmental temperatures below 25°C (21, 22). Others include non-evidence-based practices and behavioral risk factors such as immediate bathing after birth (22–25). Obstetric complications such as premature rupture of membranes have also been shown to be risk factors for admission hypothermia (22).

Neonatal hypothermia contributes to morbidity and mortality. However, there is limited data examining this relationship from high-burden countries like Kenya. We, therefore, sought to use multi-year routinely collected data from 21 newborn units in Kenya to (i) determine the prevalence of hypothermia within study hospitals amongst admissions to newborn units on their day of birth, (ii) explore the trend of hypothermia over the study period (39 months), (iii) identify factors associated with neonatal hypothermia at admission and, (iv) explore the association between neonatal hypothermia at admission and inpatient neonatal mortality.

## Methodology

This retrospective cohort study was conducted among inborn newborns identified by local clinical teams as requiring admission to 21 Kenyan Newborn Units (NBUs) on their day of birth between January 1, 2020, and March 31, 2023. This excluded referrals in and other groups of outborns and those within other areas of the hospital such as postnatal wards. The 21 NBUs are located within 20 public first-level referral hospitals and one tertiary-level hospital, distributed across Kenya. The 21 hospitals included in the study are distributed across 15 counties extending from Nairobi to 150 km north (Central region), 60 km east (Eastern region), and 350 km west (Western region) of the capital. Each county is semi-autonomous in healthcare management as health is a devolved function in Kenya (26), and the hospitals are likely to experience slightly different weather and climatic conditions. The hospitals are part of the Clinical Information Network (CIN), which has been described in detail elsewhere (4, 27–30). The included hospitals vary in size and average monthly admissions. They provide an intermediate level of newborn care (31), including continuous positive airway pressure (CPAP). The tertiary hospital included in the study has additional capabilities for providing intensive newborn care. Ethical approval for this work was provided by KEMRI's Scientific and Ethical Review Unit (KEMRI/RES/7/3/1 SSC PROTOCOL No. 2465).

Thirteen of the 21 hospitals included in this study are part of the Newborn Essential Solutions and Technologies (NEST360) Program. This is a comprehensive initiative aimed at reducing newborn mortality in sub-Saharan Africa through the provision of essential neonatal inpatient care equipment, training, advocacy, and policy changes for increased investment in newborn healthcare (32). The NEST360 program, in operation for four years, initiated implementation in Kenya in January 2020 and extended to select implementing hospitals through July 2021. Among the initiatives of this program is the provision of training on thermal care and hypothermia prevention to both maternity and newborn unit healthcare providers, the supply of thermometers and radiant warmers, mentorships, and periodic quality improvement programs (32).

Patient data were collected and recorded in a structured electronic database using Research Electronic Data Capture (REDCap) software. Trained Health Record Information Officers entered the data from structured newborn admission record forms that are part of patient files upon discharge (4, 33). A total of 61,854 deidentified records were included in the analysis. However, data (*n* = 2,800) from three months (December 2020–February 2021) affected by a country-wide health workers’ strike were excluded from the analysis due to the atypical conditions and patterns of admissions and practice during that period. Additionally, data (*n* = 250) from eight hospitals for a total of eight months (one month per hospital) were treated as missing values as they coincided with isolated hospital strike months.

We utilized daily land surface temperatures as substitutes for the room temperature in the NBU. These land surface temperatures were obtained from MODIS, a satellite source, with a spatial resolution of 1 kilometre (km) (34). Through the Geographical Positioning System (GPS), we acquired the dataset that included daily temperature recordings specifically for the geographical areas of the study hospitals. Further information and specifics regarding the dataset can be found elsewhere (34). To conduct our analysis, we calculated the average daily temperature values to derive monthly ambient temperature readings.

### Temperature measurement and definition of neonatal hypothermia at admission and its trend

Temperature measurements were conducted on the skin at multiple sites, as observed during our hospital visits and interactions with clinical teams. These measurements are typically performed at locations such as the axilla, forehead, and sternum, utilizing a range of instruments including thermometers, temperature probes, and thermal guns. The ‘documented temperature’ is the patient's body temperature as measured and recorded by a health care provider in the patient's file upon admission to the NBU.

We applied the World Health Organization's (WHO) neonatal body temperature classification as follows: >37.5°C (hyperthermia), 36.5°C–37.5°C (normal), 36.0°C–36.4°C (cold stress), 32.0°C–35.9°C (moderate hypothermia), and below 32°C (severe hypothermia) (35). In this study, we operated within the context of the WHO's warm chain protocols at the participating hospitals. While the hospitals might have had these protocols in place, including immediate drying, and skin-to-skin contact, to prevent neonatal hypothermia, we do not have specific data on their implementation during our study period, a limitation further explored in the Discussion section.

Neonatal hypothermia was defined as a newborn's body temperature below 36.0°C upon admission to the Neonatal Care Unit (NBU) (35). Monthly proportions of hypothermia were used to construct a time series plot to examine the trend over 36 months. To ensure complete data coverage, missing proportions of hypothermia for 8 months previously treated as missing were imputed using a linear mixed effects model. The resulting dataset provided average proportions of neonatal hypothermia per month for all 21 hospitals over 36 months.

### Factors associated with hypothermia at admission

To assess factors associated with hypothermia at admission, we used an ordinal logistic regression model with three ordered levels: normothermia (36.5°C–37.5°C) as the reference category, cold stress (36.0°C–36.4°C), and hypothermia (below 36.0°C, including severe hypothermia). Records with a body temperature above 37.5°C (hyperthermia, n = 2,146) were excluded from this model.

The hierarchical model incorporated the following explanatory variables: sex, birthweight, APGAR score at 5 min after birth, multiple gestations, weekday or weekend admission, the period the hospital has been part of Clinical Information Network (4), and implementing the NEST360 Program (32).

Within the Clinical Information Network (CIN), hospitals received regular feedback on various aspects, including the quality of care and the utilization of data for audit and feedback purposes. While we did not have specific audit data on hypothermia within the CIN hospitals, the CIN program employs an audit and feedback mechanism that encompasses various aspects of newborn care, including hypothermia through routine reporting. However, this does not preclude individual hospital led improvement initiatives focusing on hypothermia that may be instituted following feedback.

Two variables were included in relation to participation in the Network. The “Period in CIN” variable signified the duration of a hospital's active involvement in the CIN during the study. One group of these hospitals joined the Clinical Information Network early on between 2014 and 2016 (about 8 years or more to 2023), while the other group joined later in 2018 and 2019 (less than 8 years). Thirteen (13) hospitals had participated in the CIN for 8 or more years, while the remaining 9 hospitals had a participation period of less than 8 years. The decision to use an 8-year cut-off was based on the temporal exposure of hospitals to the Clinical Information Network (CIN) and its initiatives and interventions. Categorizing the study sites aimed to assess whether hospitals with longer exposure demonstrated different neonatal care practices and outcomes compared to those with shorter exposure.

“Study Month” variable represented the aggregated unit of calendar months used for evaluating the effects in this study. It spans from January 2020 to March 2023, encompassing the entire study period. The Study Month variable served to assess the impact and outcomes throughout this time frame. Although hospitals joined the CIN at different calendar time points, they all contributed a comparable amount of data in terms of months for this analysis. The expectation was that hospitals’ involvement in the network would lead to improved outcomes, such as a reduction in hypothermia.

The variable “NEST360 Program Implementing Site” indicated whether a hospital was actively involved as an implementing site for the NEST Program (36). Out of the total 21 hospitals included in this analysis, 13 hospitals were implementing sites for the NEST Program, while the remaining 9 hospitals were not. The NEST360 Program provided direct investments to these implementing sites, which included provision and maintenance of essential neonatal inpatient care equipment, training, mentorship, and advocacy support. As part of the program, nursing staff and other clinical care staff in the labor wards and NBUs received training. The hypothesis underlying these interventions at the implementing sites was that they would promote the adoption of enhanced care practices, leading to improved outcomes, such as a reduction in hypothermia.

Newborns with a birth weight >4,000 g, often referred to as macrosomic (37), were separately categorized from those between 2,500 and 4,000 grams to explore potential differences in body temperature patterns. Macrosomic babies, despite being generally considered at lower risk for hypothermia (23), are at a higher risk for adverse outcomes, including fatal and non-fatal incidents such as birth trauma (37). Candidate covariates with missingness above 30% were excluded from the model. Random effects were employed at the hospital level to account for clustering within hospitals.

### Effect of hypothermia at admission on neonatal mortality

We defined hospital neonatal mortality as death occurring to an NBU admission during the index admission and if the age was below 28 days (38). A binary logistic regression model was used to assess the effect of hypothermia at admission, in three categories as defined above, on neonatal mortality. The model included the following additional admission covariates: sex, birthweight, 5th minute APGAR score, temperature at admission category, mode of delivery, hypoxia (*oxygen saturation <90% or documented central cyanosis*), difficulty breathing, convulsions, lower chest wall indrawing, and severe grunting. The clinical signs, as documented in patient files, were assessed at the time of admission, concurrently with the documentation of the newborn's temperature. This simultaneous assessment makes it challenging to ascertain whether they were present before or after the detection of hypothermia. Babies with hyperthermia or those referred outward or who absconded outcomes were excluded. Cluster effects were adjusted for in the models.

All statistical analyses were performed using R Statistical Programming Software version 4.2.3 (39). Descriptive statistics were calculated on complete cases, and missing data were reported. Inferential statistics were conducted on both a complete case dataset and a dataset imputed using the Multiple Imputation by Chained Equations (MICE) package in R (40, 41). Missing data were assumed to be missing at random (MAR) (40, 41). Variables with 30% or less missingness were included in multiple imputation models, otherwise, discarded (42). Five (5) separate estimates of imputed datasets were created and later pooled to get an overall set of parameter estimates in a final single dataset for analysis (42). Complete case analysis and multiply imputed results were similar and so only complete case analysis results are presented.

## Results

A total of 61,854 inborn newborn admissions on the day of birth were recorded at 21 hospitals’ Neonatal Care Units (NBUs) in Kenya between January 2020 and March 2023. After excluding 3,050 admissions made during strike periods, a total of 58,804 admissions remained for further analysis. The hospitals, due to their varying sizes, contributed different numbers of admissions to the dataset (Table 1). In terms of reasons for admission into the Newborn Units, common criteria across hospitals include a birth weight under 1800g, an APGAR score below 7 at 5 min, respiratory issues, convulsions, hypoglycemia, breastfeeding difficulties, and congenital anomalies. Junior medical officers typically assign diagnoses based on signs and symptoms in Newborn Units. However, the main diagnoses for the study population were as shown in Supplementary Figure S1.

**Table 1:** Distribution of the study sample by hospital and the respective admission hypothermia.

Characteristic	Hospital																				
	H1	H2	H3	H4	H5	H6	H7	H8	H9	H10	H11	H12	H13	H14	H15	H16	H17	H18	H19	H20	H21
Total inborn admissions	1,640	722	2,589	508	1,539	1,958	4,797	2,173	5,873	1,421	2,034	5,126	5,325	2,374	1,198	3,946	4,877	1,734	4,582	3,558	1,080
Inborn admissions (monthly average)	46	20	72	15	43	54	133	60	163	39	56	142	148	66	33	110	135	48	127	99	30
Admission hypothermia (average) [<36.0°C] (%)	29.0%	9.7%	12.5%	29.3%	27.2%	28.1%	44.6%	12.1%	3.2%	6.8%	29.0%	33.0%	2.5%	13.4%	25.9%	11.1%	7.7%	31.3%	19.3%	9.0%	9.6%

### Demographic and clinical characteristics of the study population

Out of the total admissions with documented sex (58,651, 99.7%), the majority were male (32,564, 56.0%). Among those with documented gestation age (53,900, 91.3%), the majority were born at term (≥ 37 weeks) (32,095, 60%). Almost all, 99.1% (58,275) had birthweight documented. Less than half of these (41%, 23,876), were classified as low birthweight (<2,500 grams) at admission.

Among all admissions, nearly all had documented outcomes (58,756, 99.92%), with 7,786 (13%) resulting in death. Most of these deaths (6,193, 80%) occurred within the first three days of admission. A total of 5,934 (76%) deaths had temperature at admission recorded, among whom 1,940 (33%) were hypothermic (Table 2).

**Table 2:** Neonatal demographic and clinical characteristics and maternal details.

Characteristic	All newborns		Newborns in hypothermia model^a^		Newborns in mortality model^b^	
	Missing, *N* (%)	*N* = 58,804	Missing, *N* (%)	*N* = 45,853	Missing, *N* (%)	*N* = 45,270
Sex, *n* (%)	153 (0.3%)		112 (0.2%)		107 (0.2%)	
Female		26,087 (44.0%)		20,289 (44.0%)		20,031 (44.0%)
* *Male		32,564 (56.0%)		25,452 (56.0%)		25,132 (56.0%)
Baby resuscitated at birth, *n* (%)	18,936 (32.2%)		13,209 (28.8%)		13,025 (28.8%)	
* *No		28,604 (72.0%)		23,477 (72.0%)		23,184 (72.0%)
* *Yes		11,264 (28.0%)		9,167 (28.0%)		9,061 (28.0%)
Gestation (weeks), *n* (%)	4,904 (8.3%)		3,205 (7.0%)		3,147 (7.0%)	
* *<28 weeks		2,140 (4.0%)		1,632 (3.8%)		1,611 (3.8%)
* *28–32 weeks		8,187 (15.0%)		6,431 (15.1%)		6,326 (15%)
* *33–36 weeks		11,478 (21.0%)		9,122 (21.1%)		8,996 (21%)
* *≥37 weeks		32,095 (60.0%)		25,463 (60.0%)		25,190 (60%)
Birthweight (grams), *n* (%)	529 (0.9%)		325 (0.7%)		321 (0.7%)	
* *<1,000 g		1,873 (3.2%)		1,403 (3.1%)		1,386 (3.1%)
* *1,000–1,499 g		4,901 (8.4%)		3,832 (8.3%)		3,770 (8.3%)
* *1,500–1,999 g		8,769 (15.1%)		6,864 (15.0%)		6,752 (15.0%)
* *2,000–2,499 g		8,333 (14.3%)		6,473 (14.0%)		6,371 (14.0%)
* *2,500–4,000 g		31,137 (53.4%)		24,367 (54.0%)		24,102 (54.0%)
* *>4,000 g		3,262 (5.6%)		2,589 (5.6%)		2,568 (5.6%)
Mode of delivery, *n* (%)	191 (0.3%)		117 (0.3%)		115 (0.3%)	
* *Spontaneous vertex (SVD)		32,033 (54.7%)		24,882 (54.0%)		24,558 (54.4%)
* *Breech		1,490 (2.5%)		1,169 (2.6%)		1,150 (2.5%)
* *Cesarean section (C/S)		25,090 (42.8%)		19,685 (42.4%)		19,447 (43.1%)
APGAR score (5th minute), *n* (%)	1,531 (2.6%)		1,030 (2.2%)		1,007 (2.2%)	
* *0–3		1,487 (3.0%)		1,085 (2.0%)		1,063 (2.4%)
* *4–6		11,146 (19.0%)		8,774 (20.0%)		8,638 (19.5%)
* *7–10		44,640 (78.0%)		34,964 (78.0%)		34,562 (78.1%)
Hypoxia, *n* (%)	1,577 (2.7%)		399 (0.9%)		390 (0.9%)	
* *No		47,794 (84.0%)		37,612 (83.0%)		37,171 (83.0%)
* *Yes		9,433 (16.0%)		7,842 (17.0%)		7,709 (17.0%)
Outcome, *n* (%)	48 (0.1%)		28 (0.01%)		0 (0.0%)	
* *Absconded		99 (0.2%)		77 (0.2%)		
* *Alive		50,282 (85.5%)		39,564 (86.3%)		39,564 (87.0%)
* *Dead		7,786 (13.3%)		5,706 (12.5%)		5,706 (13.0%)
* *Referred		589 (1.0%)		478 (1.0%)		
Length of stay (all exits), *n* (%)	118 (0.2%)		84 (0.2%)		78 (0.2%)	
* *≤3 days		26,062 (44.0%)		20,090 (44.0%)		19,819 (44.0%)
* *4–7 days		15,728 (27.0%)		12,301 (27.0%)		12,178 (27.0%)
* *>7 days		16,896 (29.0%)		13,378 (29.0%)		13,195 (29.0%)
Length of stay (alives), *n* (%)	102 (0.2%)		73 (0.2%)		69 (0.2%)	
* *≤3 days		19,844 (39.0%)		15,599 (39.0%)		15,342 (39.0%)
* *4–7 days		14,910 (29.0%)		11,674 (29.0%)		11,555 (29.0%)
* *>7 days		16,114 (32.0%)		12,773 (32.0%)		12,598 (32.0%)
Length of stay (deaths), *n* (%)	12 (0.2%)		9 (0.2%)		0 (0%)	
* *≤3 days		6,193 (80.0%)		4,477 (79.0%)		4,477 (79.0%)
* *4–7 days		809 (10.1%)		623 (11.0%)		623 (11.0%)
* *>7 days		772 (9.9%)		597 (10.0%)		597 (10.0%)
Mother's age, *n* (%)	6,669 (11.3%)		4,508 (9.8%)		4,435 (9.8%)	
* *<18 years		1,519 (2.9%)		1,170 (2.8%)		1,162 (2.8%)
* *18–35 years		46,490 (89.2%)		36,920 (89.3%)		36,457 (89.3%)
* *>35 years		4,126 (7.9%)		3,255 (7.9%)		3,216 (7.9%)

^a^ Records with hyperthermia excluded.

^b^ Records with hyperthermia, absconded, referred and missing outcome excluded.

### Prevalence of hypothermia at admission among the study population

**Figure 1:**
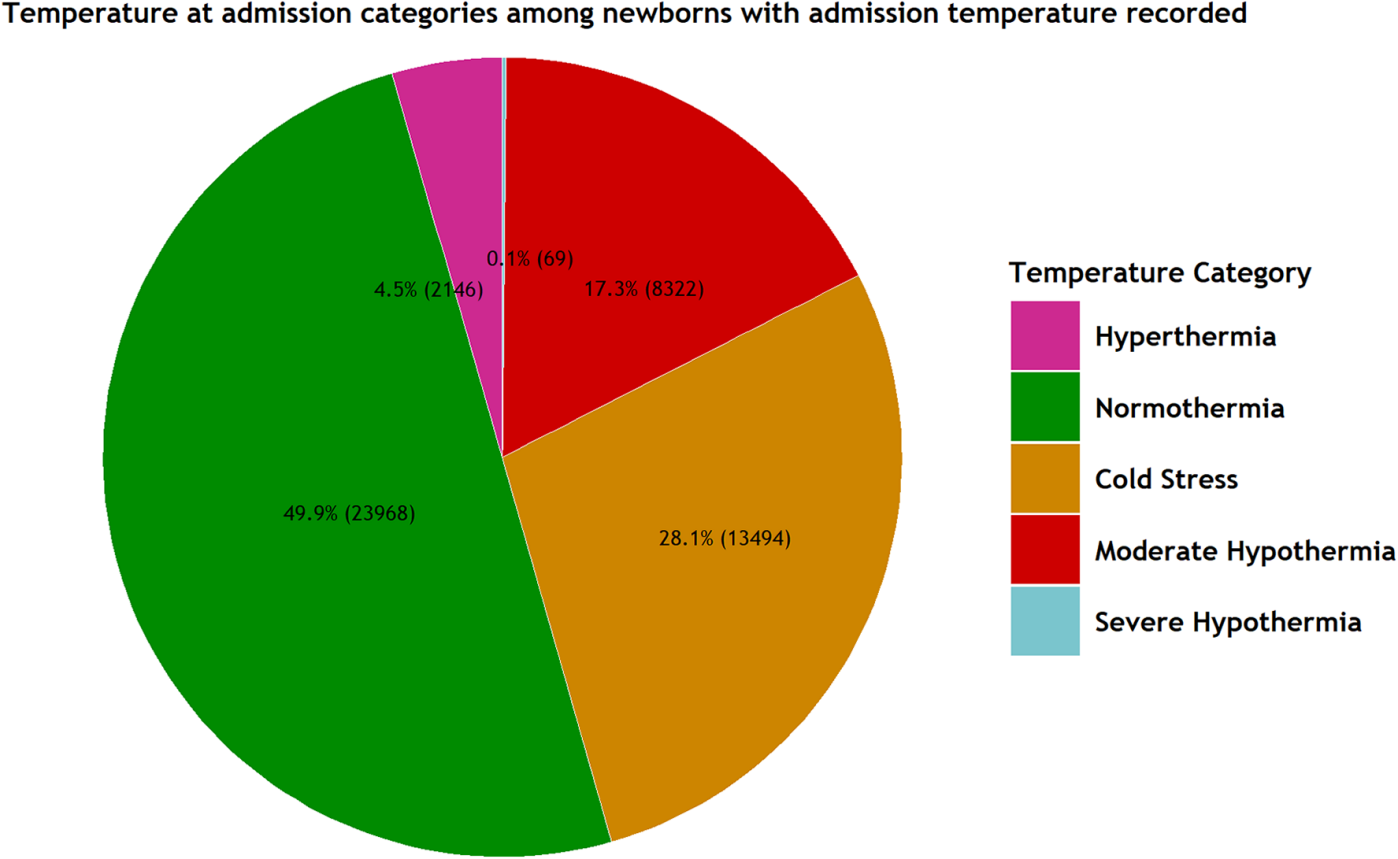
Admission diagnoses for the study population.

Out of the total admissions with the recorded temperature at admission (47,999, 82%), 8,391 (17.5%) (95% CI 17.0%, 18.0%) exhibited hypothermia (<36.0°C). The inter-hospital range for hypothermia prevalence was 3% to 45% (Figure 1 and Table 1).

### Trend and pattern of documented temperature and hypothermia

The analysis of pooled neonatal hypothermia prevalence over 36 non-strike months (January 2020 to March 2023) revealed fluctuating trends and patterns. Over the period, the prevalence fell from 20% at the start to 12% at the end (minimum monthly prevalence of 11% in February 2023 and maximum of 27% in July 2020). While it's apparent that improvements in temperature documentation occurred across all conventional birth weight groups, documentation among 2,500 g–4,000 g and >4,000 g indeed steadily improved, especially in the later months of the study. Similarly, reduction in the proportion of hypothermia appeared more consistent and relatively smoother among babies of the same weight groups (2,500 g–4,000 g and >4,000 g) in the second half of the study period compared to low birth weight groups (Supplementary Figure S2). A time series model suggests a declining linear relationship between time and prevalence of hypothermia over the 36 months (gradient of −2.37, 95% CI −3.09, −1.65).

**Figure 2:**
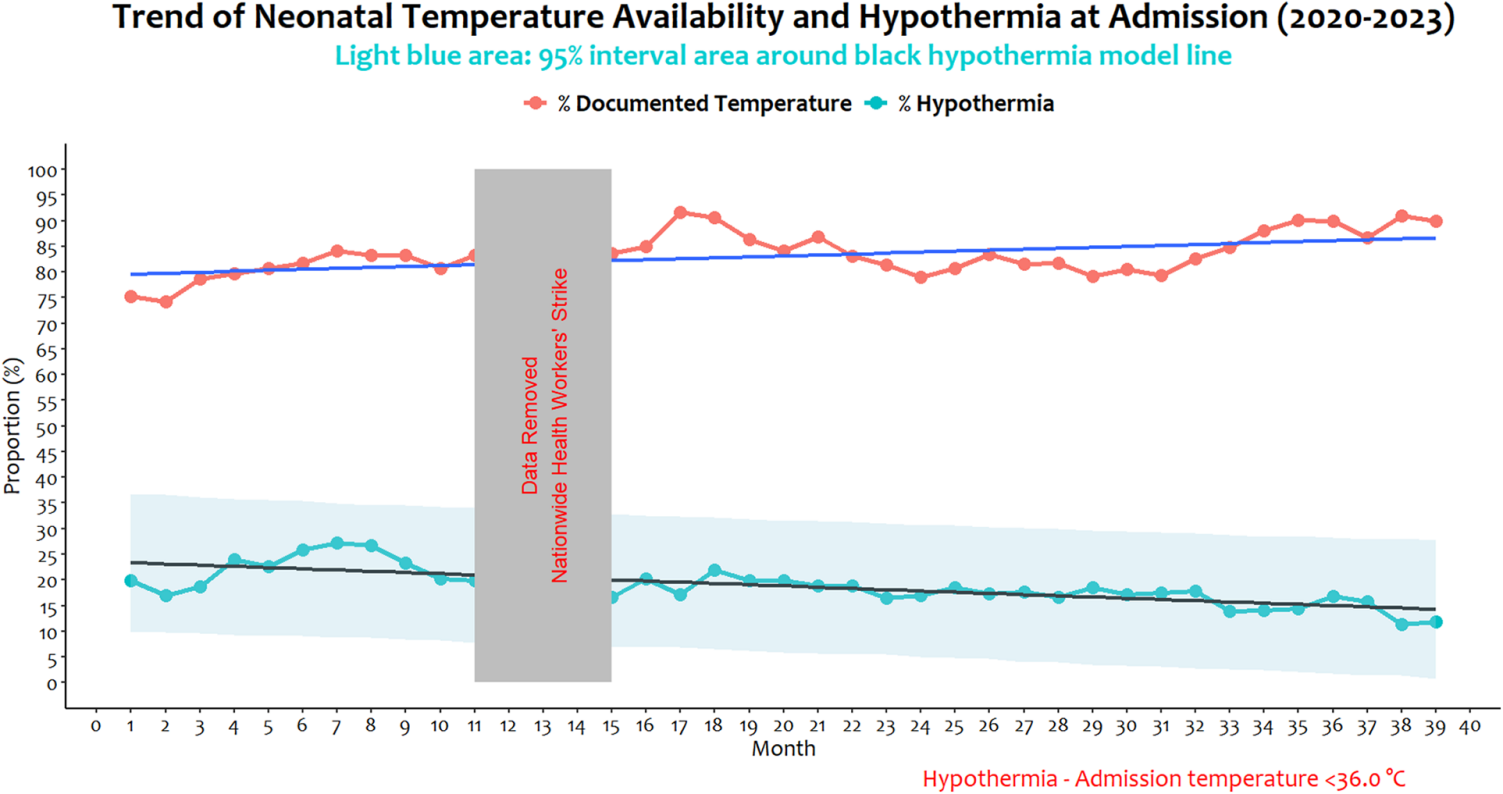
Trend of temperature documentation at admission and neonatal hypothermia (temperature <36.0°C) at admission by birth weight category between January 2020 to March 2023 excluding 3 months national strike.

Over the same period, temperature at admission documentation rose from 75% to 87% (minimum, 74%, and maximum, 91%), and with a significant increasing gradient of 1.41 (95% CI 0.60, 2.21, p = 0.0038). There was a moderate negative association between admission temperature documentation and proportions with neonatal hypothermia at admission *[correlation, r = −0.3, (95% CI −0.57, 0.02)]*, but this was not statistically significant (*p = 0.0724*) (Figure 2).

### Factors associated with hypothermia at admission

Adjusted for hospital differences, newborn's birthweight, APGAR score at 5 min, multiple gestation, weekday or weekend admission, and ambient temperature were all significantly associated with hypothermia at a newborn's admission (Table 3). Compared to newborns with normal birthweights (2,500 g–4,000 g), each of the low-birth-weight categories had increased odds for hypothermia at admission. Those below 1,000 g had the highest odds (OR 3.54, 95% CI 3.15, 3.97, *p* < 0.001) and babies born with a weight above 4,000 g had reduced odds (OR 0.87, 95% CI 0.80, 0.94, *p* = 0.001). Newborns admitted on a weekend, vs. those admitted on a weekday, had 6% reduced chances of hypothermia at admission (OR 0.94, 95% CI 0.86, 0.98, *p* = 0.003). Temperature at admission weakly correlated positively with ambient temperature (*r* = 0.02, *p* < 0.001) and for each degree centigrade increase in ambient temperature, odds of neonatal hypothermia at admission reduced by 6% (OR 0.94, 95% CI 0.90, 0.98, *p* = 0.003). Hypothermia prevalence also reduced by 1% on average with each month of participation in the network (OR 0.99, 95% CI 0.98, 1.00, *p* < 0.001).

**Table 3:** Factors associated with hypothermia at admission.

Characteristic	Hypothermia^b^, *N* (%)	Univariable analysis		Multivariable analysis	
		cOR (95% CI)	*P*-value	aOR (95% CI)	*P*-value
Sex (*n* = 45,741)^a^					
Female	3,816 (46.0)	*1.00* (*Reference)*		*1.00* (*Reference)*	
Male	4,560 (54.0)	0.98 (0.95, 1.02)	0.212	1.00 (0.98, 1.03)	0.730
Birthweight (*n* = 45,528)^a^					
2,500–4,000	3,431 (41.4)	*1.00* (*Reference)*		*1.00* (*Reference)*	
<1,000	670 (8.1)	4.47 (3.41, 5.87)	<0.001	3.54 (3.15, 3.97)	<0.001
1,000–1,499	1,226 (14.8)	2.19 (1.84, 2.6)	<0.001	2.18 (2.03, 2.34)	<0.001
1,500–1,999	1,554 (18.7)	1.49 (1.34, 1.65)	<0.001	1.56 (1.47, 1.65)	<0.001
2,000–2,499	1,198 (14.4)	1.18 (1.08, 1.28)	<0.001	1.23 (1.16, 1.30)	<0.001
>4,000	214 (2.6)	0.81 (0.73, 0.91)	<0.001	0.87 (0.80, 0.94)	0.001
APGAR score (*n* = 32,644)^a^					
7–10	5,468 (67.0)	*1.00* (*Reference)*		*1.00* (*Reference)*	
0–3	365 (4.5)	1.37 (1.26, 1.48)	<0.001	1.32 (1.27, 1.36)	<0.001
4–6	2,333 (28.5)	0.58 (0.51, 0.67)	<0.001	0.66 (0.60, 0.73)	<0.001
Multiple gestation (*n* = 45,764)^a^					
No	7,297 (87.0)	*1.00* (*Reference)*		*1.00* (*Reference)*	
Yes	1,080 (13.0)	1.14 (1.07, 1.21)	<0.001	0.91 (0.86, 0.98)	0.007
Period of admission (*n* = 45,853)^a^					
Weekday	6,215 (74.0)	*1.00* (*Reference)*		*1.00* (*Reference)*	
Weekend	2,176 (26.0)	0.93 (0.88, 0.98)	0.009	0.94 (0.90, 0.98)	0.003
Hospital level characteristics					
Ambient temperature	–	0.99 (0.98, 1.00)	0.047	0.94 (0.90, 0.98)	0.003
Study month	–	0.99 (0.98, 0.99)	0.001	0.99 (0.98, 1.00)	<0.001
NEST360 program implementing site					
Non-NEST360	–	*1.00* (*Reference)*		*1.00* (*Reference)*	
NEST360	–	1.20 (0.72, 1.99)	0.483	1.27 (0.84, 1.94)	0.260
Period in CIN					
<8 years	–	*1.00* (*Reference)*		*1.00* (*Reference)*	
≥8 years	–	0.64 (0.39, 1.06)	0.082	0.68 (0.39, 1.19)	0.170

cOR, crude odds ratio; aOR, adjusted odds ratio; CI, confidence interval.

^a^ Documented cases.

^b^ Complete cases analysis.

### Relationship between hypothermia at admission with inpatient neonatal mortality

Hypothermia at admission was found to be statistically associated with neonatal mortality after adjusting for other potential risk factors. Newborns with a temperature below 36.0°C upon admission had 35% higher odds of death (1.35, 95% CI 1.22, 1.50, *p* < 0.001) compared to those with normal temperatures (36.5°C–37.5°C). Newborns with cold stress (36.0°C−36.4°C) showed a slight, non-significant increase in the odds of death (1.05, 95% CI 0.96, 1.15, *p* = 0.29).

Newborns with low Apgar scores at 5 min, low birthweight, hypoxia, difficulty breathing, convulsions, and respiratory distress (severe lower chest wall indrawing and/or grunting) had significantly higher odds of death. Conversely, those with Apgar scores of 4–6 at 5 min after birth (OR 0.19, 95% CI 0.17, 0.22, *p* < 0.001) and birthweight above 4,000 g had a reduced odds of mortality (OR 0.64, 95% CI 0.49, 0.85, *p* = 0.002). The mode of delivery, whether breech or cesarean section (CS) compared to spontaneous vertex delivery (SVD), did not have a significant effect on mortality (*p* > 0.05) (Table 4).

**Table 4:** Factors associated with neonatal mortality.

Characteristic	Mortality, *N* (%)	Univariate analysis		Multivariable analysis	
		cOR (95% CI)	*P*-value	aOR (95% CI)	*P*-value
Sex					
Female	2,573 (45.0)	*1.00* (*Reference)*		*1.00* (*Reference)*	
Male	3,115 (55.0)	0.97 (0.94, 1.01)	0.114	1.02 (0.97, 1.08)	0.410
Thermal Category					
Normothermia	2,266 (40.0)	*1.00* (*Reference)*		*1.00* (*Reference)*	
Cold Stress	1,500 (26.0)	1.27 (1.18, 1.36)	<0.001	1.05 (0.96, 1.15)	0.290
Hypothermia^a^	1,940 (34.0)	3.19 (2.96, 3.44)	<0.001	1.35 (1.22, 1.50)	<0.001
APGAR Score					
7–10	2,532 (46.0)	*1.00* (*Reference)*		*1.00* (*Reference)*	
0–3	646 (12.0)	3.05 (2.93, 3.18)	<0.001	2.11 (1.99, 2.24)	<0.001
4–6	2,301 (42.0)	0.13 (0.12, 0.15)	<0.001	0.19 (0.17, 0.22)	<0.001
Birthweight					
2,500–4,000	1,607 (29.0)	*1.00* (*Reference)*		*1.00* (*Reference)*	
<1,000	1,140 (20.0)	64.32 (56.25, 73.55)	<0.001	39.3 (32.8, 47.1)	<0.001
1,000–1,499	1,440 (26.0)	7.94 (7.36, 8.56)	<0.001	6.38 (5.73, 7.10)	<0.001
1,500–1,999	829 (15.0)	1.76 (1.62, 1.9)	<0.001	1.89 (1.69, 2.11)	<0.001
2,000–2,499	491 (8.8)	1.11 (1.01, 1.21)	0.032	1.14 (1.01, 1.29)	0.036
>4,000	68 (1.2)	0.35 (0.28, 0.44)	<0.001	0.64 (0.49, 0.85)	0.002
Mode of delivery					
Spontaneous vertex (SVD)	3,427 (60.3)	*1.00* (*Reference)*		*1.00* (*Reference)*	
Breech	283 (5.0)	1.97 (1.74, 2.24)	<0.001	1.08 (0.88, 1.32)	0.460
Cesarean section (C/S)	1,973 (34.7)	0.65 (0.62, 0.69)	<0.001	0.99 (0.91, 1.07)	0.840
Hypoxia					
No	3,501 (62.0)	*1.00* (*Reference)*		*1.00* (*Reference)*	
Yes	2,120 (38.0)	2.49 (2.4, 2.6)	<0.001	1.59 (1.50, 1.69)	<0.001
Difficulty breathing					
No	1,274 (23.0)	*1.00* (*Reference)*		*1.00* (*Reference)*	
Yes	4,207 (77.0)	5.66 (5.32, 6.01)	<0.001	2.47 (2.25, 2.71)	<0.001
Convulsions					
No	5,107 (94.3)	*1.00* (*Reference)*		*1.00* (*Reference)*	
Yes	307 (5.7)	1.85 (1.64, 2.08)	<0.001	1.67 (1.41, 1.97)	<0.001
Indrawing					
None/mild	3,892 (72.2)	*1.00* (*Reference)*		*1.00* (*Reference)*	
Severe	1,401 (26.0)	6.82 (6.36, 7.32)	<0.001	1.77 (1.58, 1.97)	<0.001
Sternum	99 (1.8)	14.21 (10.64, 18.97)	<0.001	3.00 (1.87, 4.81)	<0.001
Grunting					
No	3,409 (64.0)	*1.00* (*Reference)*		*1.00* (*Reference)*	
Yes	1,951 (36.0)	5.14 (4.83, 5.46)	<0.001	2.21 (2.01, 2.42)	<0.001

cOR, crude odds ratio; aOR, adjusted odds ratio; CI, confidence interval.

^a^ Hypothermia, temperature below 36.0°C.

## Discussion

In this study, approximately 1 in 5 newborns experienced hypothermia upon admission at the start of the study and only 1 in 9 by the end of 3 years. Low ambient temperatures, low birthweight, low APGAR scores at birth and multiple gestation were associated with elevated odds of hypothermia. After adjusting for other neonatal co-morbidities, hypothermia at admission remained an independent factor associated with neonatal inpatient death. The population analyzed in our study consists of in-born newborns clinically determined to need admission on the day of birth, suggesting the warm chain is compromised from delivery within hospital units such as labor wards, operating theaters, and during intra-facility transfers for sick or at-risk newborns.

Our findings suggest that despite the existence of the World Health Organization's (WHO) thermal protection protocols (43), which include maintaining a warm room temperature (at least 25°C/77°F to 28°C/82.4°F), immediate drying and covering of the newborn before cord cutting, and promoting skin-to-skin contact with the mother, newborns are still being admitted into NBUs while they are hypothermic. This implies a potential gap in the application of warm chain protocols. Although we do not have data on its implementation at the study hospitals within the Clinical Information Network, the standard of care for newborns post-delivery in the hospitals generally follows the WHO's Warm Chain protocols (43). However, while these guidelines are in place, the implementation, and levels of adherence to these protocols may vary across different hospitals. The high prevalence and wide variability of newborn admission hypothermia in our study may be indicative of challenges in the implementation of these warm chain protocols. While we cannot confirm this due to our study's limitations, it underscores the need for further research to understand the potential gaps and challenges in the application and adherence to these protocols across different hospitals.

Documentation of routine care processes is often faced with a myriad of challenges, including time constraints, workload and availability of standardized forms among others (44). Across a 39 months’ study period, we note a general increase in proportion of newborns having a record of temperature at the point of admission. The 21 hospitals contributing this data are part of Clinical Information Network which promotes use of standardized admission and nurse monitoring forms, provides continuous feedback on quality of data, levels of documentation and inpatient neonatal care (28, 45). These efforts could contribute to temperature documentation improvements from 74% to 91% between January 2020 and March 2023 respectively. Better documentation might help explain reduced hypothermia prevalence if health workers early in the study period preferentially neglected to document temperatures of larger less-sick babies who are less likely to have other risk factors for hypothermia.

In the absence of such possible bias, data suggest newborns admitted to these hospitals had reduced odds of hypothermia in each extra month of participation in the network (OR 0.99, 95% CI 0.98, 1.00). This suggests that the consistent engagement and interventions associated with the Network, accumulated over an extended period, may contribute to favorable practices as has been documented for other conditions, and ultimately reduce the odds of hypothermia in newborns (46). We were not able to identify a statistically significant association between hypothermia and the continuous efforts made since 2019 to enhance Newborn Care Units (NBUs) through training initiatives, equipment provision, and the integration of quality improvement processes in 13 hospitals that are part of the NEST360 program (32). However, this may reflect limited power to detect such effects. While the NEST360 program is mainly geared towards Newborn Units, the root of admission hypothermia could lie in the labor ward. The interventions may not have sufficiently infiltrated the labor wards to achieve the required coverage and demonstrate impact. Furthermore, the warm chain interventions extend to areas like the labor ward/theatres, which fall outside the NEST360's primary focus. This mismatch could account for the observed lack of impact in our study.

Insufficient thermal protection measures after birth can result in a rapid decline in newborns’ body temperature, with rates ranging from 0.1°C to 1°C per minute (25). This leads to a drop in body temperature shortly after birth (19, 47). This phenomenon is widespread globally, as evidenced by studies reporting high prevalence rates in various countries, such as Ethiopia (66%), Nigeria (62%), Iran (85%), Zimbabwe (85%), Uganda (83%), Tanzania (22%) (14, 48–51) and South Asia (14). Despite the availability of simple, low-cost, and feasible interventions for the prevention and control of hypothermia, their underutilization and a lack of knowledge among healthcare providers regarding the impact of neonatal hypothermia on morbidity and mortality may contribute to the persistence of this widespread phenomenon (14, 25).

The transition from womb to external environment exposes newborns to lower temperatures and challenges their ability to regulate heat due to physiological and anatomical factors (25). Preventing and controlling hypothermia requires a comprehensive approach that considers environmental, physiological, behavioral, and socio-economic factors (14). We observed a significant positive correlation between newborn body temperature and ambient temperature obtained from satellite-based sources. An increase in ambient temperature by one-degree Celsius was associated with hypothermia at admission and had 6% reduced odds for hypothermia (OR 0.94, 95% CI 0.90, 0.98, *p* = 0.003). While we acknowledge that using land surface temperatures as a proxy introduces the potential for misclassification, as it may not precisely reflect the actual room temperatures within the NBUs, we hypothesized that if there were a relationship between land surface temperatures and the proportion of hypothermia upon arrival in the NBUs, it would suggest that ambient temperature fluctuations were affecting newborns within the healthcare system. This is because intra-hospital transport systems and newborn units care measures are ideally supposed to insulate newborns from external temperature variations. These findings highlight the effect of a modifiable risk factor for neonatal hypothermia and a corresponding opportunity to act and reduce it. Adhering to the WHO recommendation of maintaining a room temperature between 25°C and 28°C may potentially contribute to reducing the prevalence of hypothermia among newborns (52).

Low birthweight newborns, particularly those born preterm, have a higher susceptibility to hypothermia compared to normal birthweight infants. This risk has been widely documented (14, 25, 53). Newborns with a low (0–3) APGAR score at birth also have significantly higher odds of experiencing hypothermia at admission compared to newborns with APGAR scores of 7–10. Our study found a 32% increased odds of hypothermia in this group (95% CI 1.27, 1.36) and is consistent with data from Ethiopia and report that reduced oxygen metabolism in asphyxia may contribute to the increased odds of hypothermia (54, 55). These infants, considered to be in critical condition, require immediate medical attention. Resuscitation for extended periods can increase exposure to environmental conditions in the delivery rooms, which might not be favorable. These situations also mean babies are separated from their mothers (56). Specialized medical interventions, including thermal protection, are therefore necessary to help stabilize low very low birthweight and very sick newborns including measuring and managing their temperature to improve their chances of survival.

The relationship between neonatal hypothermia and mortality is well-established and pronounced if hypothermia is prolonged or severe (57). Our analysis demonstrated a 35% increase in the odds of death (OR 1.35, 95% CI 1.22, 1.50) in the multivariate model for newborns admitted with a temperature below 36.0°C. This is a reduction from the univariate model, where the odds ratio was 3.19. The smaller OR in the multivariate analysis suggests that hypothermia, when considered alongside confounding factors, may have a more modest but still statistically significant impact on the outcome. This association is consistent with several previous studies (16, 49, 56, 58, 59). Hypothermia is particularly dangerous for preterm infants, as they have less body fat and are less capable of generating heat to maintain their body temperature (24, 60) and is a prominent co-morbidity associated with prematurity, severe infections, and birth asphyxia, which are leading causes of neonatal mortality worldwide (56, 60).

This retrospective study has several limitations. The study design relies on existing data, which inherently restricts control over data collection and may result in missing or incomplete data. Additionally, reliance on medical records introduces the possibility of documentation errors or inconsistencies.

The study's focus on in-born newborns admitted on the day of birth may introduce selection bias and limit generalizability to other newborn populations. There were undocumented temperature records, particularly in the early months, potentially distorting the true prevalence of hypothermia. Time constraints and workload challenges among healthcare workers in the NBUs could have influenced temperature documentation accuracy and completeness.

Furthermore, the study lacked statistical significance in associating hypothermia with efforts to enhance NBUs through NEST360 Programme initiatives, possibly due to limited statistical power. More robust study designs may be needed to accurately detect such effects.

Certain unexpected findings, including the factors contributing to the wide range of hypothermia prevalence across study sites (3% to 45%), the reduced odds of hypothermia and mortality for babies with Apgar scores of 4 to 6, and the lower hypothermia rates on weekends, extend beyond the scope of our current study design. Further research is necessary to delve into and clarify these intriguing results. Additionally, the examination of warm chain practices, the use of thermometers (including the availability of low-reading thermometers) and validity of their measurements warrant prospective studies for comprehensive investigation.

Our study, with its large sample size gathered from a broad range of sites over a 39-month period, highlights the high prevalence (17.5%) of neonatal hypothermia (<36.0°C) at NBU admission. This indicates a persistent challenge in maintaining the warm chain post-delivery within hospital units and during intra-facility transfers for vulnerable newborns. Despite this globally recognized issue, we've observed a reduction over time, attributed to enhanced documentation and participation in audit and feedback improvement collaborations. Our novel approach of tracking this issue over time and longitudinal nature of our data, not commonly seen in current literature, has led to unique contributions to this field. These findings are particularly pertinent in our context, as they could catalyse the improved implementation of thermal protection protocols, ultimately leading to a decrease in the prevalence of neonatal hypothermia and other preventable morbidities.

Our research aligns with other studies on factors associated with hypothermia as well as its association with neonatal mortality, emphasizing the need for implementing existing intervention strategies. The replication of these findings using local routine inpatient care data provides a robust basis for designing interventions tailored to diverse patient profiles and environmental conditions, ensuring our strategies are both evidence-based and contextually relevant.

## Conclusion

Neonatal hypothermia emerges as a critical concern from the very moment of a newborn's birth. Its profound impact on the health of small and sick infants, coupled with its significant contribution to neonatal mortality, demands urgent attention. To confront this challenge effectively requires prioritizing essential measures from the point of birth such as immediate drying following delivery, promoting skin-to-skin contact, maintaining ambient temperatures between 25°C and 28°C, and implementing warm transportation protocols especially for the smallest and sickest babies. The strategic use of evidence based thermal care interventions, including immediate placement of hats on newborns, promoting skin-to-skin contact during transfer when practical, and the application of plastic wrap when indicated, deserve specific evaluation in routine low- and middle-income country (LMIC) settings. Utilizing routine data for assessing quality of care indicators in neonatal units is crucial for ongoing monitoring and improvement of care in low-resource settings, leading to better outcomes and reduced neonatal morbidity and mortality.

## Data Availability

The patient level datasets generated and/or analysed during the current study are not publicly available due to the primary data being owned by the hospitals and their counties with the Ministry of Health. The research staff do not have permission to share the data without further written approval from both the KEMRI-Wellcome Trust Data Governance Committee and the Facility, County or Ministry of Health as appropriate to the data request. Access applications can be made through the Data Governance Committee with details available on www.kemri-wellcome.org, or email to dgc@kemri-wellcome.org. Requests for access to primary data from quantitative research by people other than the investigators will be submitted to the KEMRI-Wellcome Trust Research Programme data governance committee as a first step through dgc@kemri-wellcome.org who will advise on the need for additional ethical review by the KEMRI Research Ethics Committee. Access to metadata and data pre-processing and analyses scripts can be made through this link: Harvard Dataverse https://doi.org/10.7910/DVN/VDSUDE).

## The Clinical Information Network Author Group

Clinical Information Network authors: the CIN author group who contributed to the network's development, data collection, data management, implementation of audit and feedback and who reviewed and approved this publication includes: Dolphine Mochache (NEST360 Kenya & Aga Khan University), Juma Vitalis (Vihiga County Hospital), Nyumbile Bonface (Kakamega County Referral Hospital), Christine Manyasi (Mbagathi County Hospital), Caren Emadau (Mama Lucy Kibaki Hospital), Charles Nzioki (Machakos County Hospital), Wagura Mwangi (Nyeri County Referral Hospital), Magdalene Kuria (Kisumu East County Hospital), Esther Njiru (Embu County Referral Hospital), Penina Mwangi (Kerugoya County Hospital), Rachel Inginia (Kitale County Hospital), Emma Namulala (Busia County Hospital), Grace Ochieng and Lydia Thuranira (Kiambu County Hospital), Felistus Makokha (Bungoma County Referral Hospital), Josephine Ojigo (Jaramogi Oginga Odinga Teaching and Referral Hospital), Beth Maina (Pumwani Maternity Hospital), Bernadette Lusweti (Thika County Referral Hospital). Amilia Ngoda (Vihiga County Referral Hospital), Dolphine Mochache (Kakamega County Referral Hospital), Jane Mbungu and Joan Baswetty (Mbagathi County Hospital), Josephine Aritho and Beatrice Njambi (Mama Lucy Kibaki Hospital), Zainab Kioni (Machakos County Referral Hospital), Lucy Kinyua (Nyeri County Referral Hospital), Alice Oguda (Kisumu East County Hospital), Loise N. Mwangi (Embu Teaching & Referral Hospital), Nancy Mburu (Kerugoya County Hospital), Celestine Muteshi (Kitale County Hospital), Salome Okisa Muyale (Busia County Hospital), Faith Mueni (Kiambu County Hospital), Rosemary Mututa (Bungoma County Referral Hospital), Joyce Oketch (Jaramogi Oginga Odinga Teaching and Referral Hospital), Orina Nyakina and Faith Njeru (Pumwani Maternity Hospital), Margaret Wanjiku Mwaura (Thika County Referral Hospital). Seline Kulubi (Bungoma County Hospital), Susan Wanjala (Busia County Hospital), Pauline Njeru (Embu County Referral Hospital), John Ollongo (JOOTRH), Samuel Soita (Kakamega County Referral Hospital), Mary Nguri (Kerugoya County Hospital), Margaret Waweru (Kiambu County Hospital), Otieno George Obop (Kisumu East County Hospital), Jeska Kuya (Kitale County Hospital), Esther Muthiani (Machakos County Referral Hospital), Esther Mwangi (Mama Lucy Kibaki Hospital), Joseph Ng’ang’a (Mbagathi District Hospital), Benjamin Tanui (Nakuru Teaching and Referral Hospital), Alfred Wanjau (Nyeri County Referral Hospital), Judith Onsongo (Pumwani Maternity Hospital),Peter Muigai (Thika County Referral Hospital) and Arnest Namayi (Vihiga County Hospital).
