# Supplementary figures and images for: Hypothermia amongst neonatal admissions in Kenya: a retrospective cohort study assessing prevalence, trends, associated factors, and its relationship with all-cause neonatal mortality
Source: Front Pediatr. 2024 Mar 27;12:1272104. doi: 10.3389/fped.2024.1272104 (PMC11004247; doi:10.3389/fped.2024.1272104)

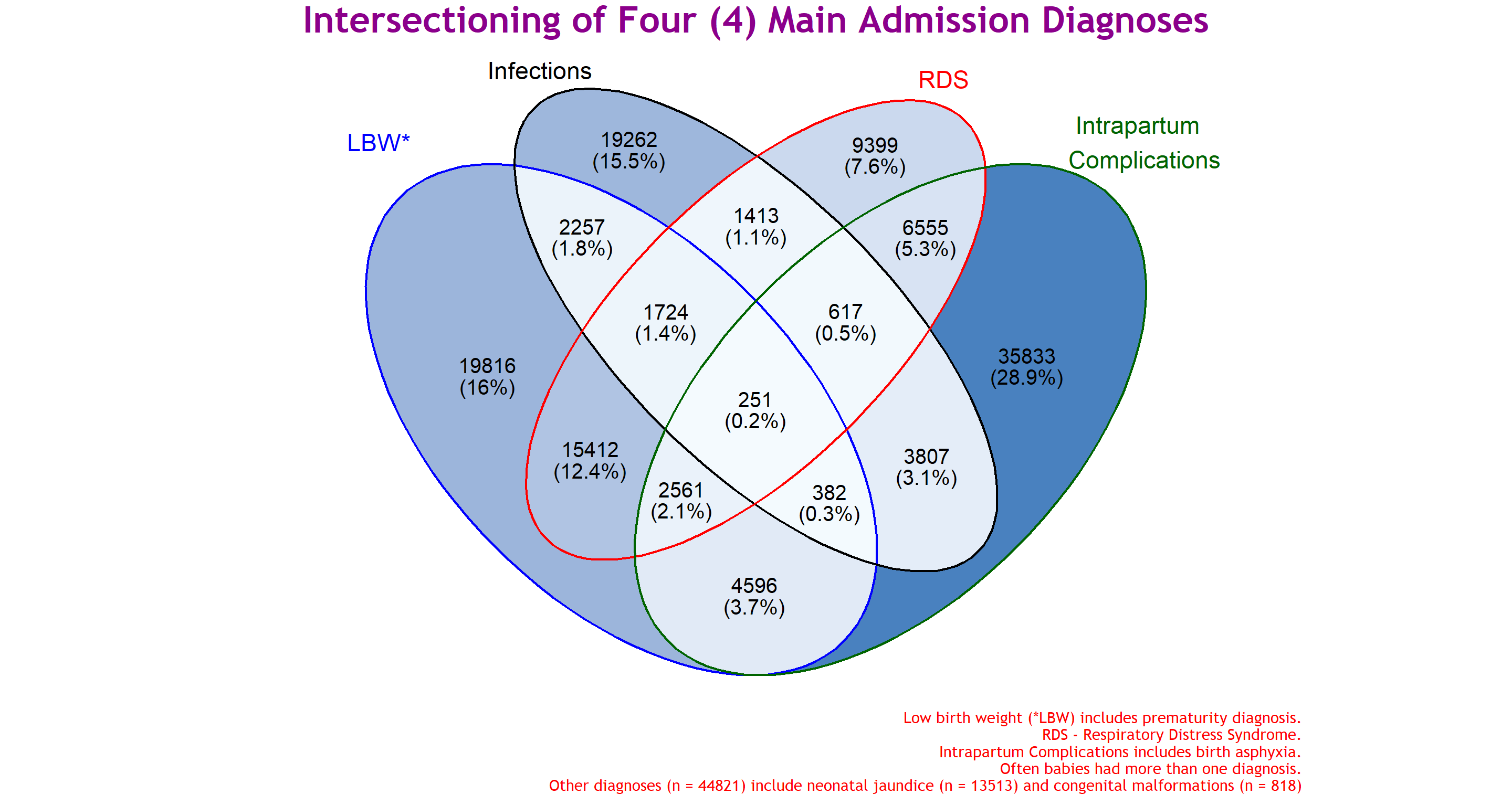

Supplement: Supplementary file 1 [file Image1.jpg]

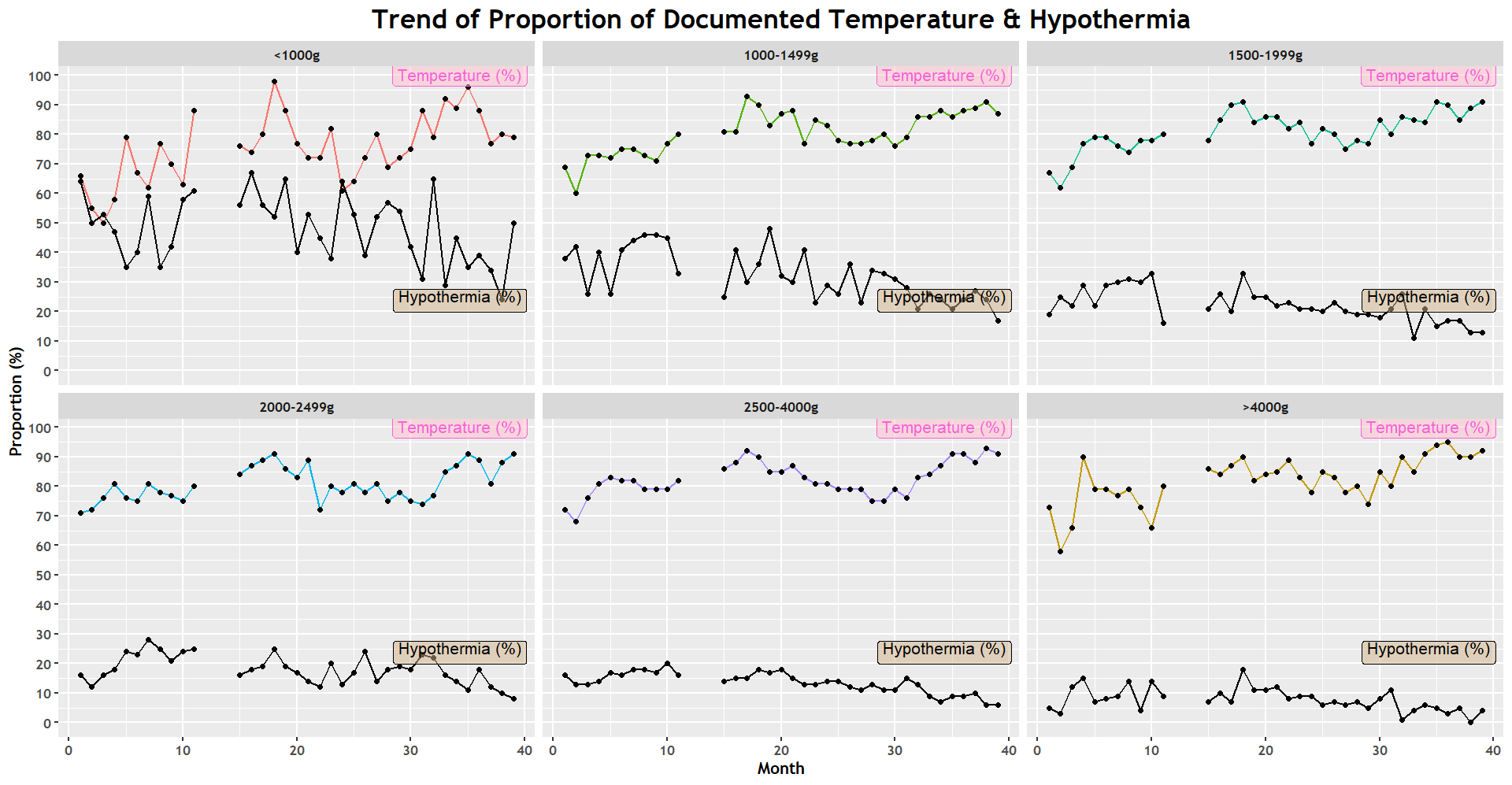

Supplement: Supplementary file 2 [file Image2.jpeg]
